# Comparison between chronic hepatitis B patients with untreated immune-tolerant phase vs. those with virological response by antivirals

**DOI:** 10.1038/s41598-019-39043-2

**Published:** 2019-02-21

**Authors:** Hye Won Lee, Seung Up Kim, Oidov Baatarkhuu, Jun Yong Park, Do Young Kim, Sang Hoon Ahn, Kwang-Hyub Han, Beom Kyung Kim

**Affiliations:** 1 Department of Internal medicine, Yonsei University College of medicine, Seoul, Republic of Korea; 2 Institute of Gastroenterology, Yonsei University College of medicine, Seoul, Republic of Korea; 3 Yonsei Liver Center, Severance Hospital, Seoul, Republic of Korea; 4 Department of Infectious Diseases, Mongolian National University of Medical Sciences, Ulaanbaatar, Mongolia

**Keywords:** Hepatitis B, Liver cancer

## Abstract

Routine nucleos(t)ide analogs (NUCs) have not yet been recommended for patients with immune-tolerant (IT) phase in chronic hepatitis B virus (HBV) infection. We aimed to evaluate prognosis of patients in untreated IT-phase (UIT group), compared to those in immune-active phase who achieved virological response by NUCs according to guidelines (VR group). Between 2006 and 2012, patients in UIT or VR groups were included. Cumulative risks of HCC and liver-related events (LREs) development were assessed. Furthermore, propensity-score was calculated based upon age, gender, diabetes and liver stiffness. UIT group (n = 126) showed younger age, lower proportion of male gender and lower LS than VR group (n = 641). UIT group had similar 10-year cumulative risks of HCC (2.7% vs. 2.9%, p = 0.704) and LRE (4.6% vs. 6.1%, p = 0.903) development, compared to VR group. When we re-defined UIT group by the lower ALT cut-offs, 10-year cumulative risks of HCC and LRE development were 2.9% and 4.8%, respectively. Using propensity-score matching and inverse probability treatment weighting analysis, similar results were reproduced. UIT group consistently had similar prognosis compared to VR group. Therefore, further large-scale prospective studies in order to verify rationales of routine NUCs in UIT group are still required.

## Introduction

Chronic hepatitis B virus (HBV) infection is a dynamic process with the interaction between viral replication and the host immune system^1,2^. Among the natural history of chronic HBV infection, “Immune-tolerant (IT)” phase is characterized by the presence of serum HBeAg, very high serum HBV-DNA level and persistently normal serum alanine aminotransferase (ALT)^2^. During this clinical phase, there is minimal or no hepatic necro-inflammation or fibrosis and the overall risk of disease progression might be low^2^. Therefore, even though many studies showed that antiviral therapy with nucleos(t)ide analogues (NUCs) might delay liver disease progression^3,4^, routine antiviral therapy for patients in IT-phase has not yet been generally recommended except for several special medical conditions, according to the current most practice guidelines^2,5–7^.

On the other hands, higher serum HBV-DNA levels are also associated with a higher risk of disease progression including development of hepatocellular carcinoma (HCC) and liver cirrhosis, even in a subgroup with normal serum ALT level^8,9^. This phenomenon might pose the question whether IT-phase patients would also benefit from earlier antiviral therapy. Furthermore, even in IT-phase patients, chromosomal HBV DNA integration and clonal hepatocyte expansion might be also found, indicating that histological inflammation, HBV-specific immune responses, and subsequent hepato-carcinogenesis might be ongoing^10–13^. From the clinical viewpoints, Kim *et al*.^14^ recently reported that untreated IT-phase patients had higher risks of developing HCC and death/transplantation, compared to immune-active phase patients treated with NUCs according to the practice guidelines. In the similar context, another study suggested the rationale of starting antiviral therapy for IT-phase patients, based upon the observation of the reduced HCC risks in the treated group^15^.

Nevertheless, so far, robust evidences favoring earlier antiviral therapy in IT-phase patients for the purpose of preventing disease progression are still lacking and whether IT-phase patients are indicated for long-term NUCs therapy still remains to be debated. Accordingly, in a real-world setting, reimbursement of antiviral therapy for untreated IT-phase patients has been still limited from the socio-economical viewpoints. Here, in the current study, we aimed to compare long-term clinical outcomes including HCC development between patients in the untreated IT-phase vs. patients in the immune-active phase who achieved virological response (VR) by NUCs therapy according to the current treatment guidelines.

## Materials and Methods

### Study subjects

Patients in the immune-active chronic hepatitis B (CHB) phase who achieved VR status by NUCs therapy (referred as VR group) according to treatment guidelines and patients in the untreated IT-phase (UIT group) at Severance Hospital, Yonsei University College of Medicine, from 2006 to 2012 were considered eligible. Chronic HBV infection was defined as positive serum hepatitis B surface antigen (HBsAg) test for at least 6 months. Inclusion criteria were as follows; (1) age ≥20 years old, (2) reliable liver stiffness (LS) value by transient elastography (TE) and (3) follow-up duration of at least 1 year. Exclusion criteria were as follows; (1) history of HCC or liver cirrhosis at the enrollment, (2) co-infection with other viral hepatitis or presence of other liver diseases, (3) current use of immunosuppressive agents, (4) HCC, hepatic decompensation or death within 6 months of enrollment and (5) other significant medical illness. If histologic information was not available, cirrhosis was clinically defined as follows: (1) platelet count <100,000/μL and ultrasonographic findings suggestive of cirrhosis, including a blunted, nodular liver edge accompanied by splenomegaly (>12 cm); or (2) esophageal or gastric varices^16^.

IT-phase was defined as serum HBV-DNA levels of ≥20,000 IU/mL, positive hepatitis B e antigen (HBeAg), and persistently normal serum ALT level during the follow-up. Serum ALT level was measured using standard laboratory procedures with the upper limit of normal set at 40 U/mL. Antiviral therapy was initiated on the basis of the treatment guidelines developed by the Korean Association for the Study of the Liver^6^ and the reimbursement guidelines of the national health insurance program of the Republic of Korea (Supplementary Table 1). VR was defined as achievement of serum HBV-DNA < 2,000 IU/mL by antiviral therapy and the index date for the VR group was a timing achieving VR.

The study protocol was consistent with the ethical guidelines of the 1975 Declaration of Helsinki and was approved by the institutional review board of Severance Hospital (No. 1-2016-0022). The informed consents were waived by the IRB, because this study is a retrospective study.

### Clinical evaluation and follow-up

During follow-up, all patients received laboratory tests including routine blood chemistry tests, serum HBV-DNA level, and other serologic viral markers every 3–6 months and underwent periodic surveillance with ultrasonography and serum alpha-fetoprotein levels to screen for HCC and portal hypertension-related complications every 6 months. In case of virological breakthrough (defined as >1 log_10_ IU/mL increase in serum HBV-DNA level from nadir on two consecutive tests) or genotypic mutation during antiviral therapy, rescue therapy was applied, if appropriate^6^.

LS values were determined using TE (FibroScan^®^, EchoSens, Paris, France) at the time of enrollment for the UIT group and at the time of VR for the VR group. The principles of LS measurement have been described previously^17,18^. Only LS values with at least 10 valid measurements, a success rate of at least 60%, and an interquartile range-to-median ratio <30% were considered reliable.

### Study end-points

The primary end-points were the development of HCC or comprehensive liver related events (LRE), which included HCC, decompensation (hepatic encephalopathy, ascites, variceal bleeding, spontaneous bacterial peritonitis, or hepatorenal syndrome), or liver-related mortality. To avoid statistical repetition in the event that a patient experienced different types of LREs at different times, we selected the first LRE for statistical analysis. HCC was diagnosed based on histological evidence or radiological findings determined by dynamic computed tomography and/or magnetic resonance imaging (nodule >1 cm with arterial hyper-vascularity and portal/delayed-phase washout)^19,20^.

### Statistical analysis

Differences among continuous and categorical variables were examined for statistical significance with Student’s *t-*test (or Mann-Whitney test, if appropriate) and chi-squared test (or Fisher’s exact test, if appropriate). A two-sided *P* value < 0.05 was considered to indicate statistical significance. Cumulative risks of HCC or LRE development were calculated using Kaplan-Meier method and compared with log-rank test.

Furthermore, to reduce the effect of selection bias and potential confounders between the UIT and the VR group, differences in the baseline characteristics were adjusted through propensity score (PS)-matching analysis and inverse probability treatment weighting (IPTW) analysis. PS was calculated using logistic regression.

All statistical analyses were conducted using IBM® SPSS® Statistics Version 23.0 (IBM Corporation in Amonk, NY, US), the SAS software, version 9.2 (SAS Institute) and R (V.3.0, http://cran.r-project.org/). Two-sided p-values < 0.05 were considered to indicate statistical significance.

## Results

### Baseline characteristics of the study population

The primary study population comprised 126 patients in the UIT group and 641 patients in the VR group. Baseline characteristics of two groups are summarized and compared in Table 1. The mean age was 52.5 years old, and 61.4% (n = 471) were male. Among the UIT group, the mean serum HBV-DNA level was 6.9 log_10_ IU/mL and 76.2% (n = 96) had serum HBV-DNA level of >6 log_10_ IU/mL. Among the VR group, 66.8% (n = 428) belonged to HBeAg-positive CHB, whereas 33.2% (n = 213) belonged to HBeAg-negative CHB. All patients in the VR group continued NUC therapy even after the achievement of VR. NUCs initially administered for the VR group comprised lamivudine (n = 269, 42.0%), entecavir (n = 217, 33.9%), adefovir (n = 77, 12.0%), telbivudine (n = 38, 5.9%), tenofovir (n = 24, 3.7%), and clevudine (n = 16, 2.5%).

**Table 1:** Comparison of baseline characteristics between two groups.

Variables	Total	UIT group	VR group	*P* value
	n = 767	n = 126	n = 641	
Age, years	52.5 ± 11.0	47.7 ± 11.1	53.5 ± 10.7	< 0.001
Male gender, %	61.4	49.2	63.8	0.002
Diabetes, %	7.8	4.8	8.4	0.162
HBV-DNA, log_10_ IU/mL	3.3 ± 1.9	6.9 ± 2.0	2.7 ± 0.9	<0.001
Platelet count, ×10^3^/uL	198 ± 65	198 ± 87	197 ± 60	0.909
ALT, U/mL	24.6 ± 9.9	23.4 ± 7.8	24.9 ± 10.2	0.128
Total bilirubin, mg/dL	0.8 ± 0.3	0.7 ± 0.3	0.8 ± 0.3	0.011
Prothrombin time, INR	0.8 ± 0.4	0.7 ± 0.5	0.8 ± 0.4	0.001
Liver stiffness values, kPa	7.3 ± 6.0	5.7 ± 2.4	7.7 ± 6.5	0.001

Data are expressed as mean ± standard deviation, or %.

Abbreviations: UIT, untreated immune-tolerant; VR, virological response; HBV, hepatitis B virus; ALT, alanine aminotransferase; INR. international normalized ratio.

Compared to the VR group, the UIT group was younger (mean age 47.7 vs. 53.5 years; p < 0.001), had the lower proportion of male gender (49.2 vs. 63.8%; p = 0.002) and the lower mean total bilirubin level (0.7 vs. 0.8 mg/dL; p = 0.011), the lower mean prothrombin time level (international normalized ratio 0.7 vs. 0.8; p = 0.001), and the lower mean LS value (5.7 vs. 7.7 kPa; p = 0.001). Furthermore, 1.6% among the UIT group had the subclinical cirrhotic LS value (defined as ≥13 kPa)^21^, whereas 8.7% among the VR group did (p = 0.003).

### Clinical outcomes in the UIT and the VR groups

**Figure 1:**
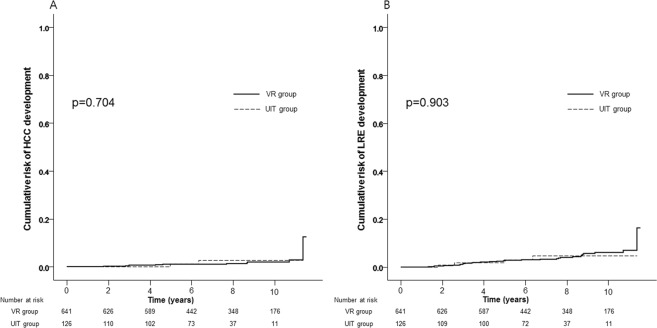
Cumulative risks of HCC (**A**) and LRE (**B**) development between the UIT and the VR groups.

Among entire population, 13 (1.7%) developed HCC and 34 (4.4%) experienced LREs during the follow-up period (median 96.6 months). Baseline characteristics were compared according to HCC development (Table 2). Patients with HCC was significantly older (mean age 60.0 vs. 52.4 years; p = 0.014) and had the higher proportion of diabetes (23.1% vs. 7.6%; p = 0.039), compared to those without HCC. The cumulative risks of HCC were similar between the UIT and the VR groups (1.1% and 2.7% vs. 1.0% and 2.9% at 5- and 10-years, respectively; p = 0.704) (Fig. 1A). After adjusting well-known prognostic variables (i.e. age, gender, presence of diabetes and LS values), adjusted hazard ratio (HR) of the UIT group (vs. the VR group) to predict HCC risk was 2.327 (95% CI 0.475–11.391; p = 0.297).

**Table 2:** Comparisons of baseline characteristics between patients who developed HCC or not and between patients who developed LRE or not.

Variables	Patients with HCC	Patients without HCC	*P* value	Patients with LRE	Patients without LRE	*P* value
	n = 13	n = 754		n = 34	n = 733	
Age, years	60.0 ± 8.2	52.4 ± 11.0	0.014	60.7 ± 8.8	52.2 ± 10.9	<0.001
Male gender, %	76.9	61.1	0.246	61.8	61.4	0.965
Diabetes, %	23.1	7.6	0.039	26.5	7.0	<0.001
HBV DNA, log_10_ IU/mL	3.4 ± 1.1	3.3 ± 2.0	0.959	3.3 ± 1.6	3.3 ± 2.0	0.905
HBeAg positive, %	46.2	72.7	0.034	47.1	73.4	0.001
Platelet count, ×10^3^/uL	193 ± 60	198 ± 65	0.786	191 ± 73	198 ± 65	0.560
ALT, U/mL	30.0 ± 6.6	24.5 ± 9.9	0.054	27.7 ± 8.9	24.5 ± 9.9	0.067
Total bilirubin, mg/dL	0.8 ± 0.4	0.8 ± 0.3	0.551	0.8 ± 0.3	0.8 ± 0.3	0.826
Prothrombin time, INR	0.9 ± 0.3	0.8 ± 0.4	0.186	0.9 ± 0.3	0.8 ± 0.4	0.036
Liver stiffness values, kPa	7.6 ± 2.3	7.3 ± 6.1	0.878	6.8 ± 2.6	7.4 ± 6.2	0.621

Data are expressed as mean ± standard deviation, or %.

Abbreviations: HCC, hepatocellular carcinoma; LRE, liver-related event; HBV, hepatitis B virus; ALT, alanine aminotransferase; INR. international normalized ratio INR, international normalized ratio.

Baseline characteristics were compared according to LRE development (Table 2). Patients with LRE was significantly older (mean age 61 vs. 52 years; p < 0.001), had the higher proportion of diabetes (26.5% vs. 7.0%; p < 0.001) and the higher prothrombin time (mean international normalized ratio 0.9 vs. 0,8; p = 0.036), compared to those without HCC. In addition, the cumulative risks of LRE development was also similar between the UIT and the VR groups (3.0% and 4.6% vs. 2.6% and 6.1% at 5- and 10-years, respectively; p = 0.903) (Fig. 1B). After adjusting well-known prognostic variables (i.e. age, gender, presence of diabetes and LS values), adjusted HR of the UIT group (vs. the VR group) to predict LRE risk was 1.341 (95% CI 0.457–3.933; p = 0.593).

### PS matching analysis

**Figure 2:**
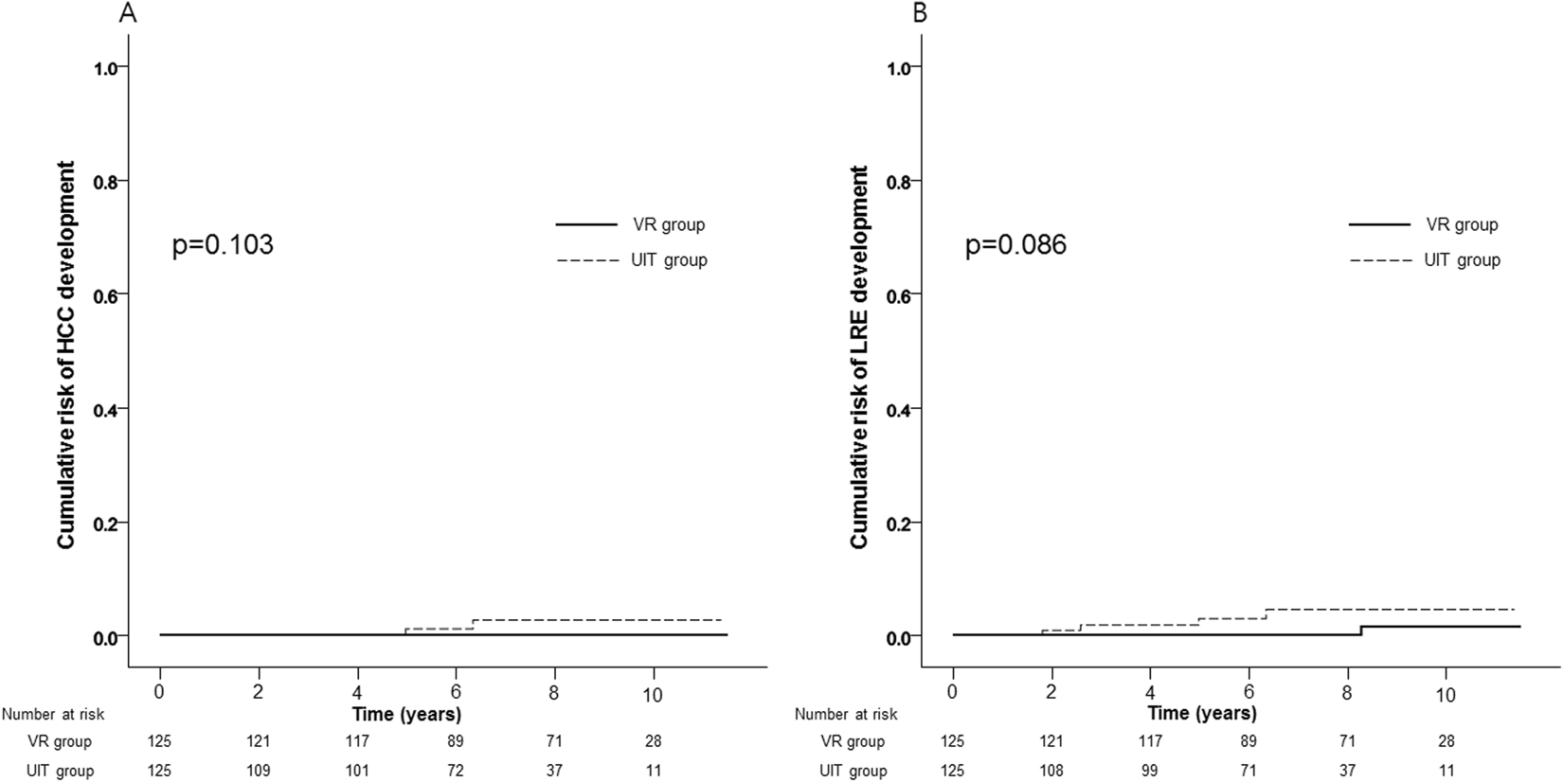
Cumulative risks of HCC (**A**) and LRE (**B**) development between the UIT and the VR groups after PS matching.

The variables used to calculate PS were as follows; age, gender, presence of diabetes and LS values. PS matching with 1:1 ratio generated 125 pairs. The UIT (n = 125) and the VR group (n = 125) showed similar baseline clinical characteristics in terms of age, gender, proportion of diabetes, body mass index, platelet count, alanine aminotransferase, total bilirubin, prothrombin time, and LS value (Table 3). After PS matching, similar results between the UIT and the VR groups were also maintained; there was no difference in the cumulative risks of HCC development (1.2% and 2.7% vs. 0.0% and 0.0% at 5- and 10-years, respectively; p = 0.103) (Fig. 2A). In terms of HCC development, during the follow-up period (median 88.4 months), two cases were observed in the UIT group, whereas none was observed in the VR group.

**Table 3:** Comparison of baseline characteristics between two groups after PS matching.

Variables	UIT group	VR group	*P* value
Age, years	47.8 ± 11.2	47.2 ± 10.2	0.636
Male gender, %	49.6	53.6	0.527
Diabetes, %	4.8	4.8	1.000
Platelet count, ×10^3^/uL	198 ± 87	216 ± 67	0.070
ALT, U/mL	23.4 ± 7.8	23.0 ± 10.0	0.735
Total bilirubin, mg/dL	0.7 ± 0.3	0.8 ± 0.3	0.138
Prothrombin time, INR	0.6 ± 0.5	0.7 ± 0.4	0.211
Liver stiffness values, kPa	5.7 ± 2.4	5.9 ± 2.3	0.656

Data are expressed as mean ± standard deviation, or %.

Abbreviations: PS, propensity score; UIT, untreated immune-tolerant; VR, virological response; HBV, hepatitis B virus; ALT, alanine aminotransferase; INR. international normalized ratio.

Likewise, after PS matching, there was no difference in the cumulative risks of LRE development between the UIT and the VR groups (3.0% and 4.6% vs. 0.0% and 1.5% at 5- and 10-years, respectively; p = 0.086) **(**Fig. 2B). In terms of LRE development, two cases (two HCCs) were observed in the UIT group, whereas 1 case (varix bleeding) was observed in the VR group.

### IPTW analysis

Furthermore, IPTW analysis was performed using PS of patients at baseline. In IPTW, each individual was weighted by the inverse probability of their basal phase, and the baseline characteristics of the UIT group and the VR group were also well balanced. In this analysis, similar results between the UIT group and the VR group were also maintained; there was no difference in the cumulative risks of HCC development (2.7% and 5.9% vs. 0.9% and 1.8% at 5- and 10-years, respectively; p = 0.068) (Supplementary Fig. 1A). Likewise, there was no difference in the cumulative risks of LRE development (4.4% and 7.5% vs. 2.5% and 5.8% at 5- and 10-years, respectively; p = 0.437) (Supplementary Fig. 1B).

### Subgroup analyses among the UIT group

**Figure 3:**
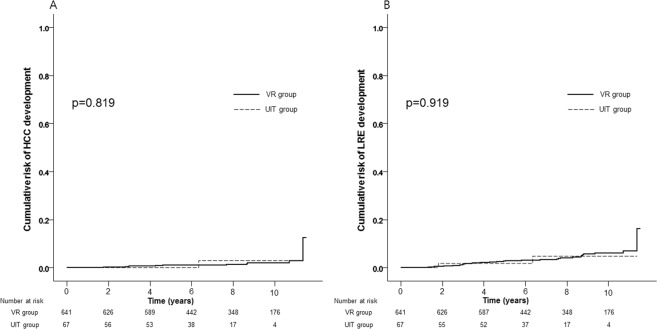
Cumulative risks of HCC (**A**) and LRE (**B**) development between the UIT group re-defined using the lower serum ALT level (<30 U/L for males and <19 U/L for females) and the VR group.

Aamong the UIT group, we performed a subgroup analysis using the lower serum ALT cut-off values (<30 U/L for males and <19 U/L for females) according to the criteria of the American Association for the Study of Liver Diseases (AASLD)^2^. Among this subgroup (n = 67), the cumulative risks of HCC development at 5- and 10-years are 0.0% and 2.9%, respectively, with no significant statistical difference compared to the VR group (p = 0.819, Fig. 3A). Likewise, the cumulative risks of LRE development at 5- and 10-years are 1.8% and 4.8%, respectively, with no significant statistical difference compared to the VR group (p = 0.919, Fig. 3B).

Furthermore, among the UIT group, we performed a subgroup analysis according to the higher serum HBV-DNA level (>1,000,000 IU/mL)^22,23^. Notably, no HCC case occurred in this subgroup with serum HBV-DNA level >1,000,000 IU/mL (n = 96) during the follow-up with a significant statistical difference, when compared to those with the intermediate range of serum HBV-DNA level (20,000~1,000,000 IU/mL) (p = 0.019). Furthermore, this subgroup showed the trend toward the lower risk of LRE development, compared to those with the intermediate range of serum HBV-DNA level (2.5% and 2.5% vs. 4.3% and 10% at 5- and 10-years; p = 0.276).

Last, among the UIT group, we performed a subgroup analysis according to the age of 40 years. Notably, no HCC or LRE occurred during the follow-up among this subgroup with age ≤40 years (n = 39). Thus, even though not statistically significant, those with age >40 years showed the trend toward the higher risk of HCC (1.6% vs. 0.0%; p = 0.379) and LRE (4.3% vs. 0.0% at 7-years; p = 0.191) at 7-years, compared to this subgroup with age ≤40 years.

## Discussion

To date, there has been the lack of evidence that NUCs might be beneficial in terms of improving overall prognosis in untreated IT-phase patients. However, there are opposing perspectives. First, normal serum ALT level does not always indicate minimal or no hepatic inflammation and disease may progress with active HBV replication, despite persistently normal serum ALT level^8,9,24^. Recently, Kim *et al*.^14^ reported the worse prognosis in the untreated IT-phase than in treated immune-active phase and another study, albeit from a small sample size, showed the reduced HCC risks in the treated IT-phase patients compared to the untreated IT-phase patients^15^. Accordingly, the European Association for the Study of the Liver guideline recommends that NUCs “may be” considered for HBeAg-positive patients with high serum HBV-DNA level and normal serum ALT level, in case of age of >30 years^1^. However, the evidence level was still only “III” with “weaker” recommendation, indicating that this thesis may be quite subject to variability depending upon future investigations. Here, in order to provide the more definite answer for this controversial issue, we conducted a comparative study between the UIT group and VR group by NUCs according to practice guidelines.

In the present study, the UIT group showed the overall similar prognosis in terms of HCC and LRE development compared to the VR group and we confirmed the reproducibility of these phenomenon not only from the unadjusted analysis but also from multivariate analysis, PS matching analysis and IPTW analysis, all of which were consistent with major reports that patients with untreated IT had minimal risk of disease progression^25^. Since HBeAg may act as an immune-tolerant protein that renders the virus undetectable by the host’s immune system^26^, HBV is regarded as non-autopathic to hepatocytes which is the main reason for the absence of liver disease despite high levels of HBV replication^27^. Indeed, there exists either no or minimal liver inflammation or fibrosis during so called “IT-phase”^28,29^, which was also supported by a very low risk for disease progression during this phase (<0.5% per year)^30^.

For substantial discrepancies between ours and the study by Kim *et al*.^14^, we would like to raise several critical issues in that study. First, whereas we defined the IT-phase patients as those who had persistently belonged to the IT-phase during the whole follow-up, Kim *et al*.^14^ classified this group as those belonging to the IT-phase for only one year since enrollment. Since virological phases might normally change according to the interaction between host and virus as time goes by, some patients initially classified as the “untreated IT group” in that study would necessarily experience significant necro-inflammation and fibrosis in the next course, both of which have the potentials for HCC development. Given that hepatic carcinogenesis gradually occurs over a long period of time via both direct and indirect pathways, a grouping by the first “1-year” observation might be inappropriate. So, as Chu *et al*.^22^ indicated, the “untreated IT group” in that study^14^ might have included immune-active patients who were in remission state after having experienced prior unrecognized necro-inflammatory events. Furthermore, since both studies are based upon each single tertiary academic hospital, some difference in the baseline characteristics may exist. Actually, the cumulative HCC risks of the UIT group among that study^14^ were 4.2% and 12.7% at 5- and 10-years, respectively, quite higher than those of ours (1.1% and 2.7%, respectively). Furthermore, the cumulative HCC risk of the treated patients among that study^14^ were 1.6% and 6.1% at 5- and 10-years, respectively, also higher than those of ours (1.0% and 1.9%, respectively). So, to resolve such an inherent selection bias, the multi-centers based cohort study with the longer follow-up span should be required.

Among the UIT group, when we analyzed a subgroup with serum HBV-DNA level >1,000,000 IU/mL, no HCC occurred during the follow-up. In the similar context, the relative lower risk of LRE development among this subgroup, even though not statistically significant owing to the type II error caused by the small sample size, was shown, compared to those with the intermediate range of serum HBV-DNA level (20,000~1,000,000 IU/mL). Likewise, regarding the age criteria among the UIT group, no HCC or LRE occurred during the follow-up among a subgroup with age ≤40 years. Thus, those with age >40 years showed the trend toward the higher risk of HCC and LRE, compared to those with age ≤40 years. So, in order to clinically define “genuine” IT-phase without liver biopsy, more stringent criteria might be required.

Compared to recent two studies^14,15^, our study has several additional strengths in terms of the methodological viewpoints. First, given that the fibrotic burden, even before progression to cirrhosis, substantially influences overall clinical outcome, adjustment for fibrotic burden between groups is essential to draw accurate conclusions^16^. Our study is the first to incorporate quantitative fibrotic burden assessed by TE into a statistical analysis. It enabled not only more detailed assessments of fibrotic burden before transition to cirrhosis but also more precise comparison of overall prognosis between two groups. Analysis by matching fibrotic burden between two groups can strengthen the scientific rationale. Furthermore, in the VR group, LS value was measured at the time of VR. Through this strategy, the potential confounding effect of potential overestimation of the LS value caused by high serum ALT levels at the time of commencement of NUCs therapy could be effectively prevented^31^. Last, this study has robust statistical power, ensured by multivariate analysis, PS-matching and IPTW analysis, all of which are approaches that can overcome bias between two groups.

As HBV is a oncogenic DNA virus that may integrate randomly into the host’s hepatocyte DNA^32^, we fully acknowledge that IT-phase itself should not be translated into an equivalence of so called “safety zone” of disease progression. As significant necro-inflammation or fibrosis should not be present in IT-phase patients according to its definition^1,2^, a proper identification of IT-phase patients by more sophisticated criteria using ancillary methods should be required. To date, non-invasive approaches by either laboratory or imaging modalities have become the mainstream in assessing histological liver fibrosis. However, there has been the lack of reliable laboratory biomarkers to detect “subclinical microscopic hepatic necro-inflammation”^33–36^. Therefore, according to the physicians’ discretion, liver biopsy should be considered seriously at least in selected cases with specific host and viral factors^34,37^.

Although our study attempted to overcome the shortcomings of previous studies, several unresolved limitations exist. First, since this is the observational study, the findings were potentially subject to selection bias. However, we conducted multiple statistical strategies to adjust for differences in baseline susceptibility between two groups, confirming the consistent results. Second, in the Republic of Korea, most (>98%) CHB patients were infected with genotype C HBV through vertical transmission, both of which were associated with a higher risk of HCC development^38–40^. Thus, these results may not be generalizable for the full spectrum of the HBV-infected population, especially in other countries. Last, the relative small sample size and the small event number might be another limitations of our study. To resolve these issues, the multi-centers based cohort study with the longer follow-up span should be required.

In conclusion, the present study consistently showed overall similar cumulative risks for disease progression in the UIT group, compared to the VR group. Therefore, recent opinions favoring routine NUCs in untreated IT-phase should be further verified with more robust evidence. In addition, large-scale prospective studies on whether treated IT-phase patients are less likely to have disease progression than untreated IT-phase patients are still required.

## Supplementary information

Supplementary Dataset 1
