# Author Correction: Comparison between chronic hepatitis B patients with untreated immune-tolerant phase vs. those with virological response by antivirals

**DOI:** 10.1038/s41598-019-53776-0

**Published:** 2019-11-21

**Authors:** Hye Won Lee, Seung Up Kim, Oidov Baatarkhuu, Jun Yong Park, Do Young Kim, Sang Hoon Ahn, Kwang-Hyub Han, Beom Kyung Kim

**Affiliations:** 1 Department of Internal medicine, Yonsei University College of medicine, Seoul, Republic of Korea; 2 Institute of Gastroenterology, Yonsei University College of medicine, Seoul, Republic of Korea; 3 Yonsei Liver Center, Severance Hospital, Seoul, Republic of Korea; 4 Department of Infectious Diseases, Mongolian National University of Medical Sciences, Ulaanbaatar, Mongolia

Correction to: *Scientific Reports* 10.1038/s41598-019-39043-2, published online 21 February 2019

The original version of this Article contained a typographical error in the name of the author Oidov Baatarkhuu, which was incorrectly given as Baatarkhuu Oidov. This has now been corrected in the PDF and HTML versions of the Article, and in the accompanying Supplementary Dataset 1 file.

The author contributions section now reads:

Conception and design of the study: Beom Kyung Kim; Generation, collection, assembly, analysis and/or interpretation of data: Hye Won Lee, Beom Kyung Kim, Seung Up Kim, Oidov Baatarkhuu, Jun Yong Park, Do Young Kim, Sang Hoon Ahn, Kwang-Hyub Han; Drafting or revision of the manuscript: Hye Won Lee, Oidov Baatarkhuu, Beom Kyung Kim; Approval of the final version of the manuscript: all authors.

