# Supplementary material for: Comparison between chronic hepatitis B patients with untreated immune-tolerant phase vs. those with virological response by antivirals
Source: Sci Rep. 2019 Feb 21;9:2508. doi: 10.1038/s41598-019-39043-2 (PMC6385334; doi:10.1038/s41598-019-39043-2)

Comparison between chronic hepatitis B patients with untreated immune-tolerant phase vs. those with virological response by antivirals

Hye Won Lee[1-3], Seung Up Kim[1-3], Oidov Baatarkhuu[1,4], Jun Yong Park[1-3], Do Young Kim[1-3], Sang Hoon Ahn[1-3], Kwang-Hyub Han[1-3], and Beom Kyung Kim[1-3]

[1]Department of Internal medicine, Yonsei University College of medicine, Seoul, Republic of Korea

[2]Institute of Gastroenterology, Yonsei University College of medicine, Seoul, Republic of Korea

[3]Yonsei Liver Center, Severance Hospital, Seoul, Republic of Korea

[4]Department of Infectious Diseases, Mongolian National University of Medical Sciences, Ulaanbaatar, Mongolia

Running title: Prognosis of immune-tolerant chronic hepatitis B

Corresponding author

Beom Kyung Kim, M.D., Ph.D.

Department of Internal Medicine, Yonsei University College of Medicine

50-1 Yonsei-ro, Seodaemun–gu, Seoul, 03722, Republic of Korea

Tel: 82-2-2228-1930; Fax: 82-2-393-6884; E-mail: beomkkim@yuhs.ac

Supplementary Table 1. The detailed reimbursement guidelines for nucleos(t)ide analogs therapy by the national health insurance program in the Republic of Korea

Clinical status	Criteria for reimbursement of nucleos(t)ide analogs therapy
HBeAg-positive CHB	Serum HBV-DNA ≥ 20,000 IU/mL and serum AST or ALT ≥ 80 IU/mL
HBeAg-negative CHB	Serum HBV-DNA ≥ 2,000 IU/mL and serum AST or ALT ≥ 80 IU/mL
Cirrhosis	Serum HBV-DNA ≥ 2,000 IU/mL
Hepatocellular carcinoma or decompensated cirrhosis	Detectable serum HBV-DNA

Abbreviations; CHB, chronic hepatitis B, HBV, hepatitis B virus; AST, aspartate aminotransferase; ALT, alanine aminotransferase

Supplementary Figure 1. Cumulative risks of HCC (A) and LRE (B) development between the UIT and the VR groups by IPTW

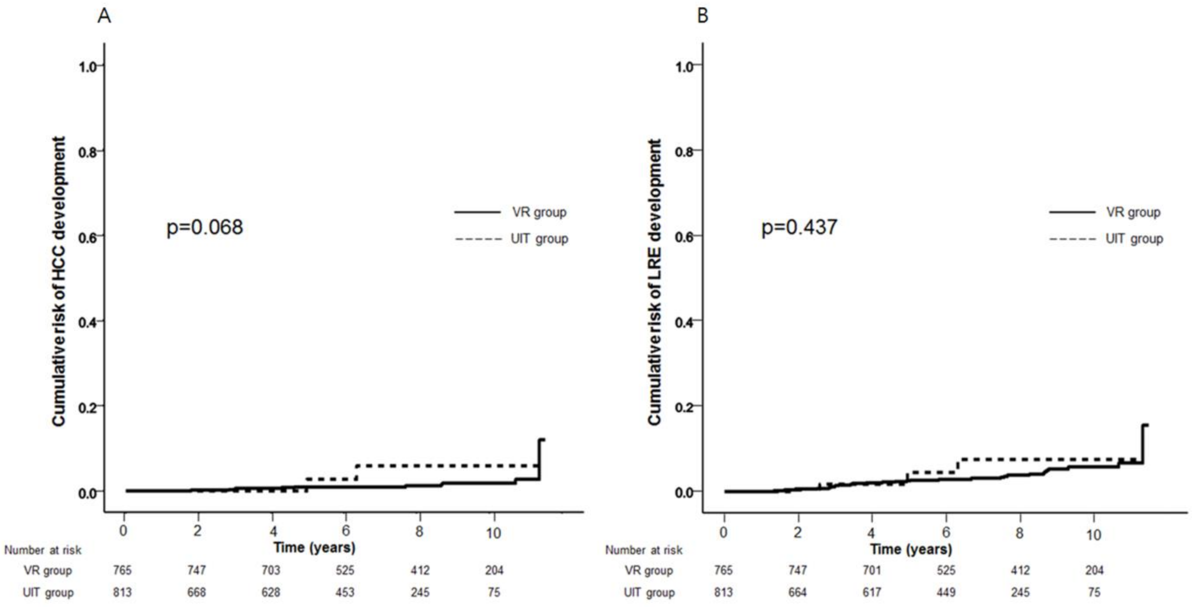

Supplement: Supplementary file 1 — Supplementary Dataset 1 [file 41598_2019_39043_MOESM1_ESM.pdf]
